# Diagnosis of human fascioliasis in Arusha region, northern Tanzania by microscopy and clinical manifestations in patients

**DOI:** 10.1186/s12879-015-1326-9

**Published:** 2015-12-23

**Authors:** Abdul-Hamid Settenda Lukambagire, Deborah N. Mchaile, Mramba Nyindo

**Affiliations:** Department of Medical Parasitology and Entomology, Kilimanjaro Christian Medical University College, P.O. Box 2240, Moshi, Tanzania; Department of Paediatrics and Child Health, Kilimanjaro Christian Medical Centre, P.O. Box 3010, Moshi, Tanzania

**Keywords:** “Human fascioliasis”, Diagnosis, Treatment, Tanzania

## Abstract

**Background:**

Human fascioliasis (HF) is a zoonotic disease that has been identified in many countries worldwide. This report concerns the identification and clinical management of cases of human fascioliasis in the suburbs of Arusha city, northern Tanzania in 2013. Fascioliasis is included among the WHO’s Neglected Tropical Diseases as a plant transmitted trematode infection. Human fascioliasis has not been described before in the East Africa region, including Tanzania.

**Methods:**

Patients presenting at a primary healthcare centre in Arusha Region, northern Tanzania provided fresh stool samples for routine ova and parasite screening (saline and iodine preparations). Subsequent stool samples were preserved in 5 % formalin in saline and subjected to ether sedimentation for examination.

**Results:**

Out of 1460 patients, 305 (21 %) were diagnosed positive for fascioliasis based on the demonstration of brownish, oval eggs with inconspicuous opercula in stool. Two distinct egg sizes were identified; large 170–212.5 by 115–150 μm (mean 194.5 by 130.5 μm) and smaller eggs 120–150 by 87.5 – 112.5 μm (mean 138.8 by 101 μm). Clinically, patients presented with fever (39 - 40 °C) and abdominal pain. Some patients had pruritis around the mouth and their lips were swollen. 3 patients were treated and cured with single dose Triclabendazole. The remaining 302 patients were treated with Nitazoxanide and 122 (40 %) were cleared of infection with a single course. Snails of the genus *Lymnaea* were found in the surroundings.

**Conclusions:**

This report serves to remind medical professionals in East Africa that HF is a probable differential diagnosis in patients presenting with similar symptoms. It is possible to diagnose fascioliasis by light microscopy although specific antigen tests are required for confirmation. Human fascioliasis however, has not been described or reported in Tanzania before and begs further investigation.

## Background

*Fasciola hepatica and F. gigantica* are trematodes commonly referred to as the liver flukes in sheep, goats and cattle where they cause serious disease with significant economic losses to farmers. Humans become infected with the *Fasciola* by ingesting metacercariae encysted on leaves of vegetables including watercress, radishes, corn, spinach, alfalfa and broccoli [1]. Humans can also become infected with *Fasciola* metacercariae in water when drunk. Adult liver flukes are found in the bile ducts and liver parenchyma of infected human and animal hosts. *Fasciola* cannot be passed directly from one person to another [2]. Eggs passed in stool of infected hosts hatch into miracidia which need freshwater lymnaeid snail vectors to develop to sporocysts, rediae and cercariae which leave the snail intermediate host and encyst on leaves of vegetation as the infective metacercariae [1–3]. After ingestion, juvenile flukes are released from the metacercariae in the small intestine, having survived stomach acid digestion. They penetrate the intestinal wall and migrate through the abdominal cavity, the liver capsule and into the liver parenchyma. Following the penetration of the capsule the immature flukes migrate through the liver tissues for about 6–8 weeks in the case of *F. hepatica* and up to 12–14 weeks for *F. gigantica*, finally entering the bile ducts where they mature and begin egg production [3, 4].

Human fascioliasis (HF) is usually asymptomatic, though symptoms may occur early on in the infection when immature flukes migrate from the intestine through the abdominal cavity and liver. Symptoms associated with HF are generally non-specific and can be categorized as acute and chronic. Symptoms of the acute disease can start 4 to 7 days after infection characterized by fever, nausea, malaise and abdominal pain. Less often patients may present with urticaria as well as subcutaneous nodules [3, 4]. The chronic phase of the infection occurs much later, usually 12 to 16 weeks after infection, when adult flukes have settled in the bile ducts. On clinical examination a tender or non-tender hepatomegaly may be found. The symptoms of chronic disease may last for months to years after infection, usually resulting from inflammation and blockage of bile ducts. This is usually complicated by biliary colic, cholestasis or cholangitis in untreated patients [5, 6].

The rather distinct and rare ova in stool samples of various patients visiting TotalCare Medical Centre was first noticed in April 2012. At the time, it was a rather common finding to have high infection rates of *Ancylostoma duodenale*, *Giardia lamblia as* well as *Entamoeba histolytica* in stool samples examined for parasitic infections. By mid-September 2012, many patients presented with clinical symptoms suggestive of typhoid fever or intestinal worm infections. When stool from these patients was examined microscopically, eggs with unfamiliar brownish, oval appearance and inconspicuous operculum were observed; this was usually accompanied by supporting biochemical and serological findings, where analyzed.

Studies conducted within the northern Africa region showed the epidemiological picture of human fascioliasis to have changed in recent years. The number of reports of humans infected with fascioliasis has increased significantly since the 1980s and several geographical areas have been described as endemic for the disease in humans [2, 4–13].

Literature cited on HF in Africa showed that both prevalence and intensity of infection were significantly higher among girls than boys, with the highest prevalence and intensity of infection being reported in the 9–11 years age group. Women were more affected than men, but not at a significant level [9–11]. Many *Fasciola* infected children were also co-infected by other parasites [10–12]. The clinical diagnosis of fascioliasis depends largely on the microscopic examination of stool samples for ova by finding eggs with an inconspicuous operculum [1, 3, 5]. Specific coprologic antigen tests have recently been developed to determine the species of *Fasciola* in clinical and field studies [12–15]. Immunodiagnostic techniques using recombinant *Fasciola hepatica* cathepsin L 1cystine proteinase, purified cathepsin L cystine proteinase, and cathepsin L-ELISA have been used to diagnose HF especially in the pre-patent acute phase of disease [13–17]. In human infection the drug of choice is Triclabendazole because it has been noted to have the highest efficacy against both the migratory juvenile and adult stages. In many areas with scarce supply of Triclabendazole, Nitazoxanide has also been used with differing success [18]. By mid- September 2012, many patients presented with clinical symptoms suggestive of fascioliasis. Tanzania and East Africa at large have also had various reports on animal fascioliasis and its management [19].

This report concerns the clinical and parasitological findings of cases of HF at TotalCare Medical Centre, a private, primary healthcare facility located in Themi Hill, Arusha region, Tanzania. Consequent to various treatment attempts with Nitazoxanide, as of October 2013, many re-infections occurred. These cases were rather evenly spread demographically including age, sex, social-economic background and race of people living in the Themi Hill surroundings, which has a notably high, seasonal population of various snail species, particularly of the family *Lymnaeidea*.

Many local inhabitants of the area use man-made deep water wells and natural streams as a source of water for domestic consumption. During the seasonal rains, the area is subject to flooding and soggy, marshy soils especially towards the lower lying residential areas.

## Methods

### Ethical approval

This study was approved in July of 2012 by the health management team of TotalCare Medical Centre as an in-house clinical case study. In the conduct of this study, compliance to good clinical practice as defined by the Helsinki Declaration was observed. All subjects involved provided written informed consent. Children’s parents offered the consent on behalf of their children as expressed in the Declaration of Helsinki.

### Clinical

Most patients presenting at the clinic complained mainly of high grade fever (39 - 40 °C), abdominal pain at the upper right quadrant area and malaise. No abnormality was detected on abdominal examination. Few of these patients also presented with complaints of pruritis around the mouth with swelling of the lips. Jaundice was also observed. In clinical practice such presentation would range from malaria, typhoid fever to urinary tract infection (UTI). Laboratory investigations commonly ordered were blood smear for malaria parasites, Widal test for typhoid fever and urinalysis. Patients were provided with stool containers containing 5 % formalin diluted in saline as a preservative.

### Clinical management

The clinical management of patients in the beginning of this study was punctuated by apparent uncertainty and lack of conclusive laboratory diagnosis of human fascioliasis in our setting. Nevertheless, following a cautious approach and exhaustive research on fascioliasis, we came up with a reliable management protocol for patients diagnosed with human fascioliasis.

Three of the 305 patients were treated with Triclabendazole administered at 10–12 mg/kg, which cleared the patients of *Fasciola* infection. The remaining 302 were treated with Nitazoxanide at a dose of 500 mg twice daily for 6 days in adults and 200 mg twice daily for 3 days in children older than 4 years. Only 125 patients were cleared of infection.

### Laboratory diagnosis

Patients presenting at the outpatient department (OPD) were asked to provide a stool sample on day 1, 3 and 6 for the qualitative diagnosis of parasite infection. Sample collection and processing protocol was as recommended by Cheesbrough [20]. Briefly, for fresh samples stool in a clear transparent container was processed within 30 min of collection. A plastic scoop provided with the stool container was used to sample the edges and core of the specimen, collecting approximately 0.5 gm of feces. This was mixed to homogeneity in 10 microlitre saline on a glass slide. A drop of 1 % iodine was placed on the homogenized fecal preparation and a coverslip applied. Iodized preparations were quickly examined under the light microscope for identification of parasite eggs/ova at ×100 and ×400 magnification.

Subsequently, patients who received anthelminthic treatment were provided with 50 ml stool containers containing 5 ml of 5 % formalin diluted in saline as a preservative. They were instructed on how to collect the stool sample, typically a scoop full or approximately 5gm to be mixed with the preservative and the cover sealed tight. The sample was then brought to the laboratory the following day. This protocol was routinely followed and proved to be useful when patients were treated with anthelminthics to assess effect of treatment.

Briefly, the stool was mixed to homogeneity in the preservative (5 % formalin in saline). Ether (2 ml) was then added to the homogenate and mixed thoroughly. The mixture was then left to stand for 20 min before a sample of the sediment and the supernatant was collected for examination. Approximately 0.02 ml sample was placed at the centre of a microscope slide and a drop of iodine added. A cover slip was placed over the mixture, and the slide examined at x100 and x400 magnification.

A graduated reticle inserted in the objective piece was first calibrated against the stage micrometer for standardization. Egg size estimations were then made at ×100 magnification, and pictures taken at ×400 magnification for clarity of features.

Repeat stool samples were taken day 3, 6, 14 and 21 post-therapy to screen for the presence of ova or parasites. When a diagnosis of fascioliasis was tentatively reached, all samples suspected to have eggs were stored at 2-8 °C and transported to the reference laboratory at the Kilimanjaro Christian Medical University College for confirmation of the diagnosis. In cases of mixed infections, patients were first managed for other infections. Examination of stools for earlier suspected *Fasciola* eggs was started after confirmation of treatment for co-infections. This served as a further quality control method for the diagnosis arrived at.

The data was then analyzed using descriptive statistics.

## Results

The majority of patients attending the clinic (96 %) were from the Themi-Njiro area (living within 2-10 km of the clinic), in Arusha municipality, Tanzania. A few patients also came from other municipalities outside Arusha. Children below 12 years were the most often diagnosed with fascioliasis (45 %).

Blood smears were mostly negative for haemo-parasites, including malaria, but showed eosinophilia. The Widal test for typhoid fever was positive in some patients with titres of 1:160. Urinalysis was negative for bacterial infection but showed moderate bilirubinuria. Stool examination showed presence of oval *Fasciola* eggs along with *Ancylostoma duodenale * eggs or *Strongyloides stercoralis *larvae. Most of these were treated with Albendazole and effect of treatment was observed for 2 weeks after completion of medication. During this treatment period though, patients who had initially presented with urticaria worsened.

**Fig. 1:**
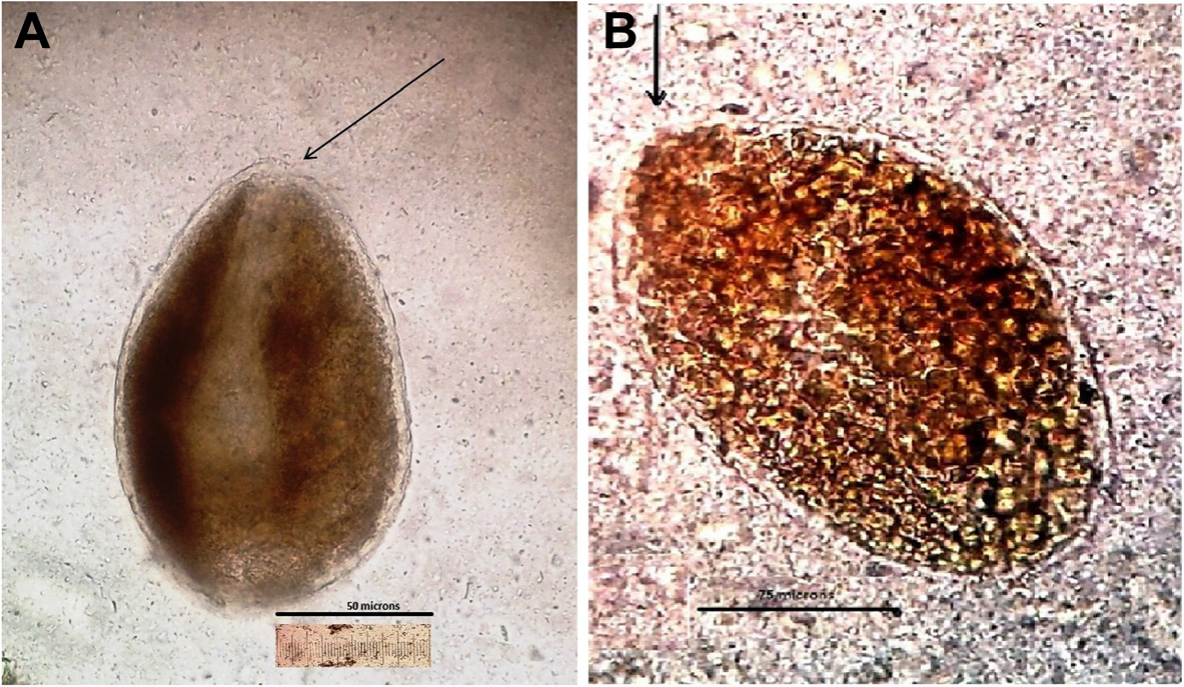
**a** and **b** Eggs of *Fasciola* (a, small size; 130.5 by 102 μm and b, large 205 by 148 μm) as seen in an iodine stained stool sample after formol-ether sedimentation. Original magnification x400 (arrow indicates position of the inconspicuous operculum; bar scale = 50 μm and 75 μm)

Oval *Fasciola* eggs (Figure 1) were demonstrated in 305 out of 1460 stool specimens. We reviewed 50 samples fixed in 5 % formalin in saline and stored for 1 week at 2-8 °C. Two distinct egg size patterns were noted; large ovoid eggs 170–212.5 by 115–150 μm (mean 194.5 by 130.5 μm) and a smaller egg range of 120–150 by 87.5 – 112.5 μm (mean 138.8 by 101 μm).

**Fig. 2:**
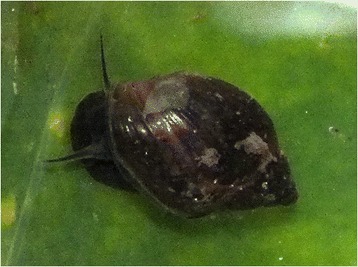
A snail of the genus Lymnaea, one of the snails which act as intermediate host of *Fasciola* on a piece of vegetation from a natural stream

**Fig. 3:**
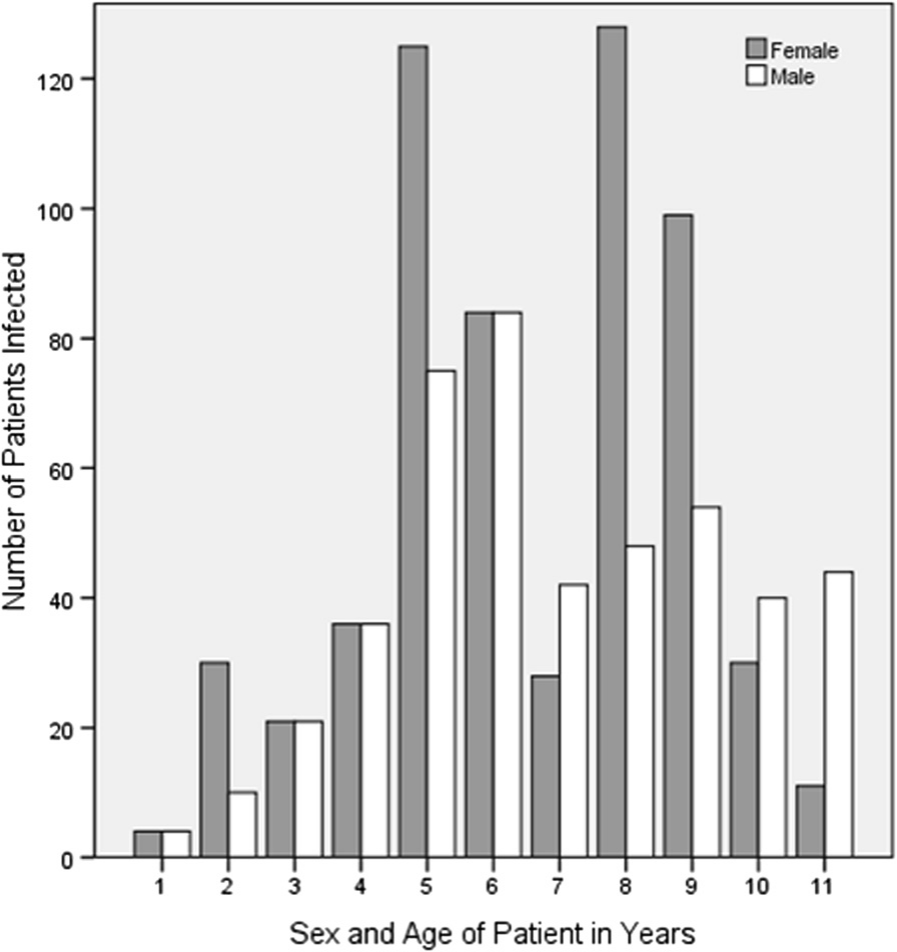
Frequency of occurrence of fascioliasis in males and females stratified from 1 to 11 years based on stool examination

**Fig. 4:**
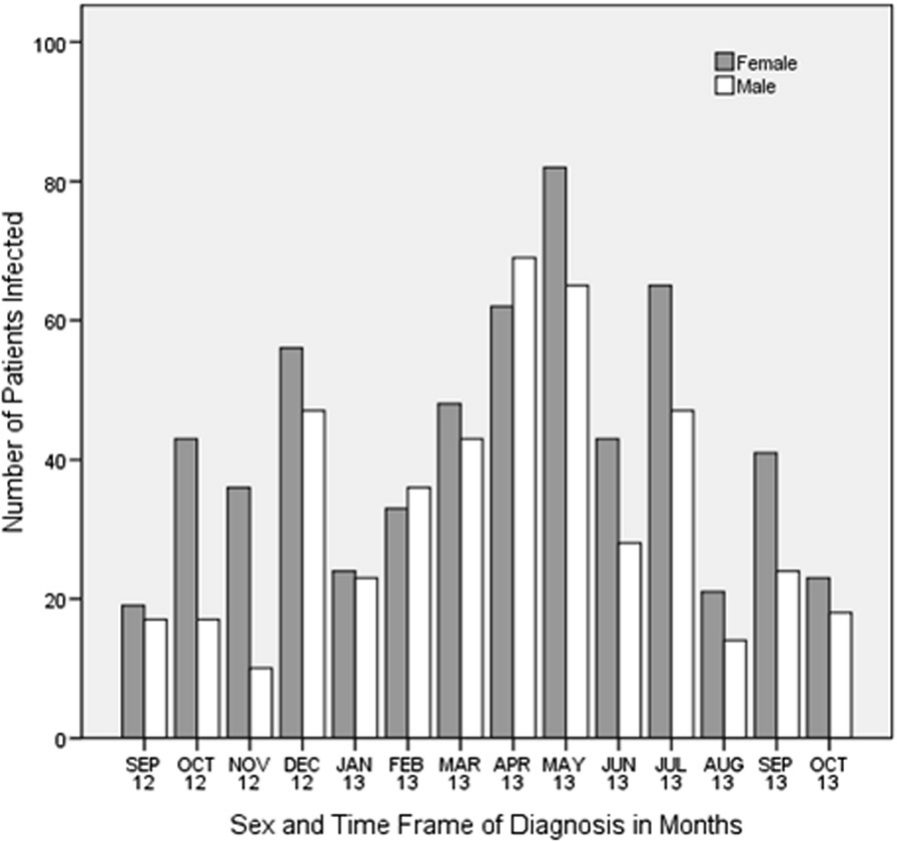
Frequency of diagnosis of fascioliasis in males and females of all age groups from September 2012 to October 2013

A snail of the genus *Lymnaea*, found in a natural water source around the study area is presented in Fig. 2. Sex and age distribution of the patients diagnosed with fascioliasis and the annual distribution of cases diagnosed at TotalCare Medical Centre are shown in Figs. 3 and 4 respectively.

The results of stool examination for *Fasciola* eggs before and after treatment as well as re-infection after treatment are given (see Flow Chart). 305 samples out of 1460 tested positive for *Fasciola* eggs in stool. Out of these 103 (34 %) were males and 202 (66 %) were females. 138 (45 %) were below 12 years old. All 3 patients treated with Triclabendazole were cured. 134 patients out of 302 returned for stool examination within a two week period after treatment with Nitazoxanide; 83 (62 %) of these were below the age of 12 years (data not shown); 122 patients were cleared of infection with single dose Nitazoxanide of whom 67 (55 %) were below 12 years old. 36 patients out of the 302 treated with Nitazoxanide manifested severe reaction to the drug which necessitated discontinuation; 25 (69 %) of these were female with only 8 (22 %) below 12 years (data not presented in the chart). After successful treatment confirmed by re-examination of stool samples for eggs over a 2 week period, 42 of the original 305 patients were re-infected with *Fasciola* at varying time intervals during the study period (12 months); 29 (69 %) of these 42 patients were children in the high risk age group i.e. below 12 years (data not shown).

**Flow Chart:**
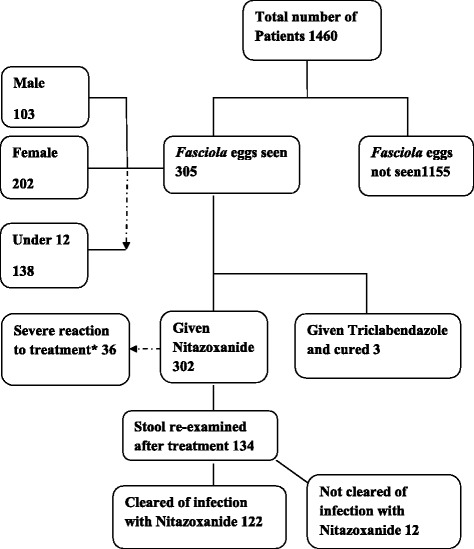
Showing Results of stool examination of 1460 stool samples for *Fasciola* eggs (pre- and post- treatment and effect of treatment). *Side effects of Nitazoxanide were noticed more frequently and severely among females than males (data not shown). All patients presented with abdominal pain (day 2–4), diarrhea (day 2–3), pharyngitis and flu-like syndrome (day 2–6) of treatment. In a few patients with pruritis, swelling and rashes of hands and feet was observed

Other laboratory procedures performed and data analysis are presented in Table 1 and Table 2 below . They included urinalysis, blood sugar, liver enzymes and cell blood count. The comment column represents significant findings

**Table 1:** Results of other laboratory procedures done

Test Procedure done and tools used	Number tested out of 305	Percent positive	Comment
Urinalysis for Bilirubin using Uriscan Optima analyser on 10SGL sticks	290	95	Bilirubin concentration varied from 0.5 – 1.0 mg/dL for most patients interpreted as mild bilirubinuria
Stool examination for protozoa including *Entamoeba histolytica*, *Giardia lamblia*. Worms including *A.duodenale*, *A. lumbricoides* and *Strongyloides stercoralis*	302	99	Of 305 samples
			Intestinal worms 140 (46 %)
			*E. histolytica* 109 (36 %)
			*G. liamblia* 55 (18 %)
Sterco-brown, loosely formed stools, fatty or mucoid with blackish exudates.	302	99	Very common

**Table 2:** Results of Hematology and Biochemistry tests done

Test Procedure done and tools used	Number tested out of 305	Recorded readings	Normal Ranges
Blood sugar test using one-touch auto test kit	198	Fasting 5.5 mmol/L	Fasting 2.0 – 4.0 mmol/L
		Random 12.0 mmol/L	Random 3.5 – 7.0 mmol/L
Liver enzymes/function tests using Statfax Chemwell auto analyser	152	AST 50–65 U/L	AST <50 U/L
		ALT 55–65 U/L	ALT < 45 U/L
		Direct Bilirubin 0.6-0.9 mg/dL	Direct Bilirubin 0.2-0.4 mg/dL
Haematology complete blood picture including Hb, levels of MCV, MCH & MCHC; using D-Cell 30 auto haematology 3 index analyser	207	Eosinophils 0.6-1.4 %	Eosinophilis <0.4 %
		Hb 9.5-11 g/dL	Hb 11–14 g/dL
		MCV 60–66 fL	MCV 70 fL
		MCH 20–26 g/dL	MCH 32.5 g/dL

* Amino transferases, were slightly elevated (normal ranges AST <46 U/L, ALT < 50 U/L)

** Direct bilirubin (normal range <0.5 md/dL) was markedly raised. *** In some patients the eosinophilia was highly marked

****The MCV and MCH concentrations were markedly below the normal range. Normal ranges for random blood sugar (3.5 – 5.5 mmol/L), Hb (11.0 – 14.0 g/dL)

## Discussion

Animal fascioliasis is a liver disease of ruminants which has been described in many parts of the world including a few African countries [1–5, 7–12]. Recently, human fascioliasis has also been described as a zoonosis acquired from domestic animals. Human fascioliasis is acquired by the ingestion of *Fasciola* metacercariae encysted on vegetables or in drinking water. The distribution of human fascioliasis has recently been reported in four African countries including; Egypt, Ethiopia, Cameroon and South Africa. In most rural African settings, domestic animals including cattle, sheep and goats are part of agricultural activities, for provision of meat, milk and hide. Similarly, in these settings, vegetables including lettuce, radishes, corncob, alfalfa, and amaranths are cultivated to provide vegetable food source. Rural and semi - rural African settings readily provide the environment suitable for the completion of the life cycle of animal fascioliasis, which includes infected animals that would release infective eggs into the environment and the subsequent development of cercariae within *Lymnaea* snails, up to development of metacercaria which encyst on vegetable leaves. When human and animal fascioliasis concur the transmission cycle of fascioliasis is amplified many fold.

The suburbs of Arusha city, where this study was conducted, provide a scenario described above for efficient transmission of animal fascioliasis to humans, i.e. the agricultural system in this area includes cattle, sheep and goats and vegetables are cultivated in abundance as an additional food source. Additionally, some of the inhabitants drink untreated water from natural streams.

In our case series, which involved 1460 persons over a 12 month period, 305 (21 %) were found to be infected with fascioliasis. Because *Fasciola* eggs are not detected during the acute infection, and because the disease has not been described before in Tanzania, it is likely that laboratory investigations intended to detect intestinal worm infections in stool would have missed the recognition of *Fasciola* eggs in the stool samples.

We also observed two distinct egg sizes on microscopic examination: large ovoid eggs mean 194.5 by 130.5 μm and smaller oval eggs mean 138.8 by 101 μm, which correspond to reported egg size ranges for fasciolid eggs [1, 5]. The eggs recovered in this study are notably wider than reported egg size ranges for both *F. hepatica and F. gigantica* from other reports [1, 21]. This could be due to preservation in 5 % formalin in saline (for 1 week) before measurements were taken. It could also be a slight variation is egg size for this region as no previous studies have reported the infection characteristics among humans, and egg sizes have been noted to vary according to geographical location [1, 5, 21]. The presence of two distinct egg sizes, sometimes recovered from stool samples of the same patient also point to possible co-infection by the different species, and may further insinuate the possibility of hybrid species [21, 22]. Although we were unable to speciate the causative parasite as being *Fasciola hepatica* or *F. gigantica* in this particular clinical study, other veterinary and malacology studies conducted in the region and East Africa in general [19, 22, 23] have shown the presence of both species. The climatic and demographic distribution of the two species has been highlighted [21–23] and of note is the presence of both species in certain geographical regions, subject to altitude and fresh water availability. Because *Fasciola* eggs have an inconspicuous operculum, it is difficult for the unsuspecting investigator to identify them with certainty, even in well documented endemic regions [1–3, 5–8].

Triclabendazole is the drug of choice for the treatment of both animal and human fascioliasis as noted in other studies [12, 18]. In this study, three patients were cleared of HF by a single dose of this drug. Use of Nitazoxanide did not clear all patients treated. This finding was consistent with some earlier reports.

This study has shown that more females than males are infected with HF and that fascioliasis is quite often associated with children below 12 years in both sexes. This finding is in agreement with what has been previously reported about the epidemiology of HF in some African and tropical countries [4, 6, 8–11]. The reasons for sex and age bias for HF could be because, at least in our settings, young girls and boys are culturally engaged as drawers of domestic water for house hold use. This practice would expose them more frequently to infection with HF than would adults. At TotalCare Medical Centre, records also indicated that there were more women and young children who visited the health centre than adult males.

Laboratory investigations including bilirubin levels indicated elevated serum and urinary bilirubin which is in agreement with findings by other studies on HF [7, 24]. Examination of blood films showed that most patients had marked eosinophilia, which is also of value in the diagnosis of HF during the acute phase of the disease [24]. Clinically, fascioliasis patients present with fever and malaise, urticaria and swelling of lips during the acute stage of disease. In this study, pain at the upper right abdominal quadrant was a common clinical finding in most of the patients whose stools were found positive for *Fasciola* eggs. Pruritis was also reported by some of the patients and swelling of the lips was also seen. The latter three findings have also been described by other studies [7] as common findings associated with HF in the chronic, patent phase of the disease.

## Conclusions

The challenges associated with a definitive diagnosis of human fascioliasis, especially at the clinical level are many: First, eggs are never passed in stool during the acute phase of the disease, therefore patients presenting clinically are likely to be missed on routine stool screening especially if only a single sample is examined without concentration. Clinical symptoms are also usually less pronounced in subsequent infections [3, 5, 24]. Much of the symptomatology presented in this study was further confounded by other infections diagnosed and cannot definitively be attributed to fascioliasis, even where eggs were found in patients’ stools.

Secondly, although we examined at least 2 samples per patient, we used only a qualitative concentration/sedimentation method as these screenings were not prejudiced towards the detection of *Fasciola* eggs alone. No quantification was done nor were any methods employed for the purpose. Other intestinal parasites were often diagnosed and consequently managed before specific screening for fascioliasis was started. Lastly, the definitive diagnosis of fascioliasis by species is usually based on the use of specific coprologic antigen tests or molecular analysis [11–15]. This study was conducted in-house in a primary health centre where neither of these diagnostic options were available, nor have they been approved for use in the country and region to date. Human fascioliasis has not been hitherto reported from Tanzania or any other East African country. Subsequent studies could focus on this distinction and make further clinical associations once the disease has been recognized as a significant public health problem.

This study serves as a reminder to the medical profession in East Africa that HF, a food borne trematode infection, is probably of more common occurrence than has hitherto been realized.

## Abbreviations

ALT: Alanine transaminase
AST: Aspartate amino tranferase
ELISA: Enzyme linked immune-sorbent assay
fL: femtolitres
g/dL: gram per decilitre
Hb: Haemoglobin
HF: Human fascioliasis
MCH: Mean corpuscular haemoglobin
MCHC: Mean corpuscular haemoglobin concentration
MEPI: Medical Education Partnership Initiative
MCV: Mean corpuscular volume
mg: milligram
NTD: Neglected tropical disease
OPD: out patient department
UTI: Urinary tract infection
U/L: unit per litre

## References

[1] Valero MA, Perez-Crespo I, Periago MV, Khoubbane M, Mas-Coma S (2009). Fluke egg characteristics for the diagnosis of human and animal fascioliasis by *Fasciola* hepatica and F. gigantica. Acta Trop.

[2] Mas-Coma MS, Esteban JG, Bargues MD (1999). Epidemiology Of Human fascioliasis: A Review And Proposed New Classification. Bull World Health Organ.

[3] Chandler AC, Read CP (1968). Trematodes or Flukes: Other Trematodes III. In: Introduction to Parasitology.

[4] Acha PN, Szyfres B. In: Sceintific and Technical Publication No. 580. Zoonoses and Communicable Diseases Common to Man and Animals. 3rd Edition; vol III Parasitoses. Pan American Health Organisation. Washington, D.C.: Pan American Health Organization; 2003. Pp 115 – 125.

[5] Cuomo MJ, Noel LB, White DB. Diagnosing Medical Parasites: A Public Health Officers Guide to Assisting Laboratory and Medical Officers USAF. 4th Edition. Air Education and Training Command Publications; 2009.

[6] Esteban JG, González C, Bargues MD, Angles R, Sánchez C, Náquira C (2003). High Fascioliasis infection in children linked to a man-made irrigation zone in Peru. Journal of Tropical Medicine and International Health.

[7] Mas-Coma S, Bargues MD, Valero MA (2008). Fascioliasis and Other Plant Borne Trematode Zoonoses. Int J Parasitol.

[8] Mas-Coma S. Epidemiology of fascioliasis in human endemic areas. J Helminthol. 2005;79(3):207–16. http://www.ncbi.nlm.nih.gov/pubmed/16153314 10.1079/joh2005296 16153314

[9] Curtale F, Hassanein YA, Barduagni P, Yousef MM, Wakeel AE, Hallaj Z, Mas-Coma. Human Fascioliasis Infection: Gender Differences within School-Age Children From Endemic Areas Of The Nile Delta, Egypt. Trans R Soc Trop Med Hyg. 2007;101(2):155-60. 10.1016/j.trstmh.2006.05.006 16890257

[10] Curtale F, Hassanein YA, El Wakeel A, Mas-Coma S, Montresore A (2008). Distribution of Human Fascioliasis by Age and Gender Among Rural Population in the Nile Delta, Egypt. Trans R Soc Trop Med Hyg.

[11] Fentie T, Erqou S, Gedefaw M, Desta A (2013). Epidemiology Of Human Fascioliasis And Intestinal Parasitosis Among Schoolchildren In Lake Tana Basin, Northwest Ethiopia. Trans R Soc Trop Med Hyg.

[12] Zumaquero-Rı’os JL, Sarracent-Pe’rez J, Rojas-Garcı’a R, Rojas-Rivero L, Martı’nez-Tovilla Y, Valero MA (2013). Fascioliasis and Intestinal Parasitoses Affecting School Children in Atlixco, Puebla State, Mexico: Epidemiology and Treatment with Nitazoxanide. PLoS Negl Trop Dis.

[13] O’Neil SM, Parkinson M, Dowd AJ, Strauss W W, Angels R, Dalton JP (1999). Short report: Immunodiagnosis of human fascioliais using recombinant *Fasciola* hepatica cathepsin L 1cystein proteinase. Am J Trop Med Hyg.

[14] O’Neil SM, Parkinson M, Dowd AJ, Strauss W, Angels R, Dalton JP (1998). Immunodiagnosis of *Fasciola* hepatica infection (fascioliasis) in a human population in the Bolivian Altiplano using purified cathepsin L cystein protienase. Am J Trop Med Hyg.

[15] Rokni MB, Massoud J, O’Neil SM, Parkinson M, Angels R, Dalton JP (2002). Diagnosis of human fascioliasis in the Gilan province of Northern Iran: Application of cathepsin L-ELISA. Diagn Microbiol Infect Dis.

[16] Strauss W, O’Neil SM, Parkinson M, Angels R (1999). Dalton JP Short report: Diagnosis of human fascioliasis: detection of anti-cathepsin L antibodies in blood samples collected on filter papers. Am J Trop Med Hyg.

[17] Santana BG, Dalton JP, Camargo FV, Parkinson M (2013). The Diagnosis of human fascioliasis by Enzyme-Linked Immunosorbent Assay (ELISA) Using Recombinant Cathepsin L Protease. PLoS Negl Trop Dis.

[18] Fairweather I, Boray JC (2009). Fasciolicides: Efficacy, Actions, Resistance and Its Management. J Vet Pathol.

[19] Keyyu JD, Kyvsgaard NC, Monrad J, Kassuku AA (2009). Effectiveness of strategic anthelmintic treatments in the control of gastrointestinal nematodes and *Fasciola* gigantica in cattle in Iringa region. Tanzania Tropical Animal Health Production.

[20] Cheesbrough M (2011). District Laboratory Practice in Tropical Countries.

[21] Mas-Coma S, Bargues MD, Valero MA (2014). Diagnosis of human fascioliasis by stool and blood techniques: update for the present global scenario. Parasitology.

[22] Howell AH, Mugisha L, Davies J, LaCourse EJ, Claridge J, Williams DJ (2012). Bovine fasciolosis at increasing altitudes: Parasitological and malacological sampling on the slopes of Mount Elgon, Uganda. Parasites & Vectors.

[23] Swai ES, Ulicky E (2009). An evaluation of the economic losses resulting from condemnation of cattle livers and loss of carcass weight due to Fasciolosis: a case study from Hai town abattoir, Kilimanjaro region. Tanzania Livestock Research for Rural Development.

[24] Hakyemez IN, Aktas G, Savli H, Kucukbayrak A, Gurel S, Tas TA (2012). Fascioliasis Case: a not Rare Cause of Hypereosinophilia in Developing Countries, Present in Developed too. Mediterranean Journal of Hematological Infectious Diseases.

